# Diagnostic Value of Diffusion Weighted Magnetic Resonance Imaging in Evaluation of Metastatic Neck Lymph Nodes in Head and Neck Cancer: A Sample of Iranian Patient

**DOI:** 10.31557/APJCP.2019.20.6.1789

**Published:** 2019

**Authors:** Fatemeh Alamolhoda, Fariborz Faeghi, Mohsen Bakhshandeh, Aslan Ahmadi, Morteza Sanei Taheri, Sahar Abbasi

**Affiliations:** 1 *Department of Radiology Technology, School of Allied Medical Sciences, *; 3 *Department of Radiology, Shohada-E-Tajrish Hospital, Shahid Beheshti University of Medical Sciences,*; 2 *Ear Nose Throat (ENT) and Head and Neck Surgery Research Center, Hazrat Rasoul Akram Hospital, Tehran University of Medical Sciences, Tehran, Iran.*

**Keywords:** DWI, MRI, neck lymph nodes, Head and neck cancer, metastasis

## Abstract

**Objective:**

To evaluate the diagnostic value of DWI in assessment of metastatic neck lymph node in a sample of Iranian patients with Head and Neck cancer.

**Methods:**

25 patients with 80 neck lymph nodes were analyzed using 1.5 T MRI. DWI was performed with b values of 0 and 1,000 s/mm^2^. Short axis diameter and ADC values (min, max and mean) were calculated for metastatic and non-metastatic neck LNs and results were compared with histopathological findings. The optimal ADC thresholds were analyzed using receiver coefficient characteristic (ROC) curves for discriminating between metastatic and benign neck lymph nodes.

**Result:**

Histopathological findings revealed that there are 45% (n=36) metastatic and 55% (n=44) non-metastatic neck LNs respectively. There was no statistically significant difference in short axis diameter between the two groups (p = 0.346). However, The ADC values of metastatic neck LNs were significantly lower than those of non-metastatic neck LNs (p < 0.001); 0.90 ± 0.10 × 10^-3^ mm^2^/s vs 1.06 ± 0.12 × 10^-3^ mm^2^/s ( ADC mean ), 0.78 ± 0.08 × 10^-3^ mm^2^/s vs 0.92 ± 0.20× 10^-3^ mm^2^/s ( ADC min ) and 1.02 ± 0.12 × 10^-3^ mm^2^/s vs 1.24 ± 0.15 × 10^-3^ mm^2^/s (ADC max ). The optimal mean ADC threshold value was equal to 0.996 × 10^-3^ mm^2^/s for differentiating malignant from benign lymph nodes with sensitivity, specificity and accuracy of 80.56 %, 77.27 % and 71.59 % respectively.

**Conclusion:**

MR diffusion imaging and ADC values as a non-invasive technique can assess metastatic neck LNs in head and neck cancer with higher sensitivity, specificity and accuracy.

## Introduction

Head and neck cancer is placed on the fifth most common cancer worldwide, accounting for about 3% to 4% of all malignancies. Lymph node metastases are found in more than half of these patients and demonstrate a most important factor in predicting outcome and selecting treatment (Vandecaveye et al., 2012; Noij et al., 2015; Dudau et al., 2014; Kim et al., 2009; Mack et al., 2008). It has been reported that the presence of a single metastatic node decreases the rate of survival by 50% and the rate of affecting to bilateral disease by an additional 50% (Dudau et al., 2014). Differentiation of benign and malignant neck lymph nodes is important especially in the presence of head and neck malignancy for staging, treatment planning and follow-up of cancer (Vandecaveye et al., 2009; de Bondt et al., 2009; Ali, 2012; Dawood et al., 2014; ElSaid et al., 2014; Ali and El Hariri, 2017). Small lymph nodes with a maximum short axial diameter below 10 mm represent one of the most challenging problems for radiologists (de Bondt et al., 2009; ElSaid et al., 2014). Conventional imaging including: Ultrasound (US), computed tomography (CT) and magnetic resonance imaging (MRI) relies on the morphological criteria of the lymph node including maximum short axis diameter, lymph node hilum loss and necrosis along with heterogeneous pattern of post contrast enhancement and perinodal infiltration. These image methods can diagnose cervical lymphadenopathy, but, they cannot differentiate benign lymph nodes from malignant ones accurately ( Ali, 2012; Dawood et al., 2014; and El Hariri, 2017; Razek et al., 2006; Holzapfel et al., 2009; Salem et al., 2014; Liang et al., 2017). Another imaging modalities such as single photon emission computed tomography (SPECT) and positron emission tomography (PET) can be helpful in this differentiation but they are expensive and are limited to low spatial resolution, as well as false diagnosis of physiological and inflammatory lymph nodes( Ali, 2012; Ali and El Hariri, 2017; Razek et al., 2006; Holzapfel et al., 2009). Ultrasound guided fine needle aspiration cytology (USgFNAC) can help in characterizing cervical lymph nodes, but this method is invasive with false negative results as this is dependent on the operator ( Ali, 2012; Ali and El Hariri, 2017; Razek et al., 2006; Holzapfel et al., 2009; Liang et al., 2017).

Diffusion-weighted MRI (DWI) is a non-invasive technique that provides quantitative information of the molecular diffusion in several compartments. Any architectural changes in the proportion of extracellular to intracellular water protons like metastases in lymph nodes will alter the apparent diffusion coefficient of the tissue. So, DWI provides information for identification and characterization of different tissues and lesions (de Bondt et al., 2009; Dawood et al., 2014; ElSaid et al., 2014; Perrone et al., 2011; Zhang et al., 2013; Ragheb et al., 2014). The aim of this study is to evaluate the diagnostic value of DWI in assessment of metastatic neck lymph node in a sample of Iranian patients with Head and Neck cancer.

## Materials and Methods

*Patients*

This study was conducted prospectively from 3 May 2017 to 10 January 2018 at the MRI department of Shohadaye Tajrish Hospital, and was included 25 consecutive patients with histologically proven Head and neck cancer and with enlarged neck nodes. According to the histopathological diagnoses, the patients were included as 9 patients with larynx cancer, 6 patients with Thyroid cancer, 4 patients with pharynx cancer, 3 patients with tongue cancer and 3 patients with parotid cancer. None of these patients had received neck clearance surgery, biopsy, chemotherapy or radiotherapy for neck LNs before MRI examination. Patients’ age ranged between 17-77 years old and the affected side was located in the left side in 3 patients, in the right side in 3 patients and there was bilateral affection in 19 cases. After an MRI examination (lasting for 2 weeks from 2 to 15 days) all patients underwent neck lymph node surgical and received a definite pathological diagnosis. In total, 80 lymph nodes, 44 benign nodes, and 36 metastatic nodes were analyzed in this study. The study was approved by the local ethical committee of the university, and informed consent was obtained. 

*MRI Examination*

Neck MRI examination were performed using a 1.5-T system (Magneto Avanto Siemens, Erlangen Germany). All patients were scanned with dedicated head and neck coils in the standard supine position. 

*The MR protocol is as follows*

• T2W-FSE-sagittal: TR: 3200 ms, TE: 80 ms, slice thickness: 4.5 mm, Matrix: 320×256, NEX: 2

• STIR-coronal: TR: 4500 ms, TE: 40 ms, TI: 160 ms, slice thickness: 4.5 mm, matrix: 256×256, NEX: 2

• T2W-FSE-axial: TR: 4350 ms, TE: 83 ms, slice thickness: 5 mm, Matrix: 320×256, NEX: 3

• T1W-FSE-axial: TR: 520 ms, TE: 18 ms, slice thickness: 5 mm, Matrix: 320×256, NEX: 3

• T2W-axial-fat sat: TR: 3500 ms, TE: 80 ms, slice thickness: 5 mm, Matrix: 320×256, NEX:3 

Acquisition of diffusion-weighted images using single shot echo planar imaging (EPI) sequence was achieved in the axial plane. Diffusion gradient encoding was done in the three orthogonal planes (X, Y, Z) with two b values b=0 and b=1,000 s/mm^2^. Imaging parameters for DWI were as follows: TR= 4400 ms, TE= 88 ms, FA=90, Matrix size=192×192, slice thickness = 4 mm, NEX=6, bandwidth= 1446 Hz/pixel. The ADC map was calculated automatically using a standard software imager in the MR system. The total scan time of DWI lasts 2 minutes. In addition, contrast-enhanced MRI scan is provided in sagittal, coronal and axial planes for each patient in order to confirm the localization of malignant tissue as well as any cysts or necrosis. 

*Image analysis*

All MR images were read independently by two radiologists experienced in the head and neck MRI interpretation who did not any previous knowledge regarding the histopathological type of the nodes. 

Conventional MR images were assessed for the evaluating morphological features of each lymph node. We selected the LNs with short axis larger than 5 mm.

The final number of neck lymph nodes that were selected in the analysis was equal to 80 lymph nodes. In the case of patient with multiple lymph nodes (LNs), everyone was evaluated separately. The assessment of nodal ADC value includes drawing a region of interest (ROI) with greatest diameter for covering the pathologic node in all sections in which it was present and averaging the results. In this study, we excluded the necrotic areas from analysis to avoid presenting a false high ADC. All the ADC values were averaged from three-time measurement and were expressed as the mean ± standard deviation. Three ADC values (min ADC, max ADC, mean ADC) were calculated for all selected LNs. 

Finally, MRI results were compared with the results of the pathological examination of the neck dissection samples.

*Statistical Analysis*

Statistical analysis of data was performed considering special characteristic (including apparent diffusion coefficient (ADC) value of the malignant tumors and benign lesions in head and neck cancer) in Statistical Package for Social Science (SPSS) version 25. 

Descriptive Statistics was performed to display prevalence of the malignant and benign lesion in males and females and also to demonstrate mean age of them. The homogeneity of gender and age was tested between groups through chi-square independence test and independent T-test, respectively. Because of non-normal data distribution, the Mann-Whitney test was performed to compare average ADC values (ADCmin, ADCmax, and ADCmean) between malignant and benign tumors in patients with head and neck cancer. And a value of p<0.05 was considered significant. 

Three receiver operating characteristic (ROC) were curved to evaluate diagnostic capability of ADC values (ADCmin, ADCmax, and ADCmean) and to determine the cut-off value for differentiating malignant from benign nodes.

**Table 1:** Descriptive Statistics of Age and Gender

Variable	Benign	Malignant	P-value
Gender			
Male (%)	5 (41.7%)	9 (69.2%)	0.165*
Female (%)	7 (58.3%)	4 (30.8%)	
Mean Age (years)	12 (51.70 ± 1.73)	13 (46.97 ± 2.31)	0.099**

*, Chi-Square Independent Test; **, Independent T-Test

**Table 2:** Pairwise Comparison of Average ADC (×10^-3^ mm^2^/s) Values between Groups

Group	mean ADC a	min ADC a	max ADC a
Benign	1.06 ± 0.12*	0.92 ± 0.20	1.24 ± 0.15
Malignant	0.90 ± 0.10	0.78 ± 0.08	1.02 ± 0.12
P-value	<0.001	0.001	<0.001

* mean ± Standard Deviation, a, ×10^-3^ mm^2^/s

**Figure 1:**
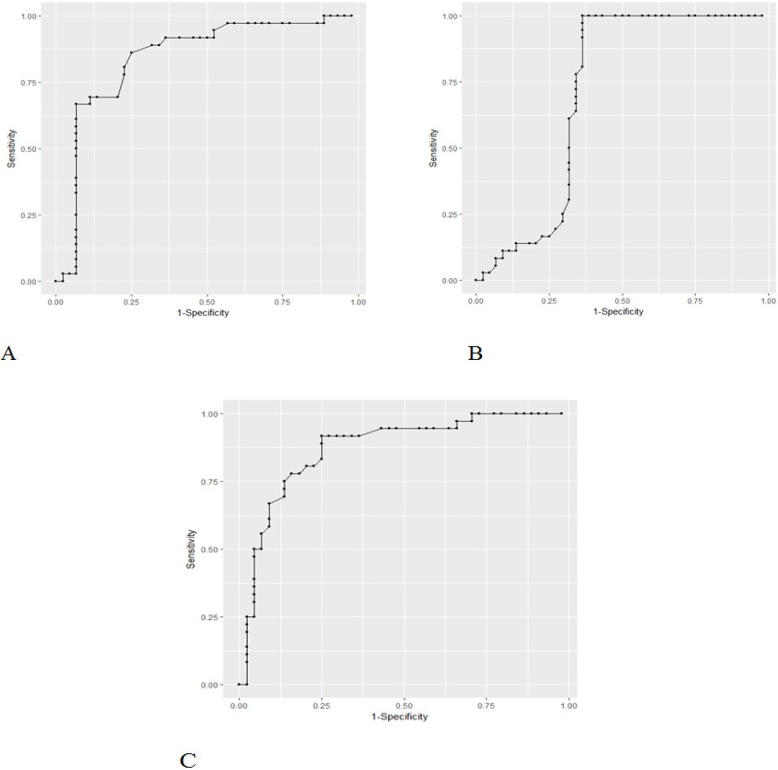
ROC Curve for the A, ADCmean values; B, ADCmin; C, ADCmax for discrimination of malignant nodes from benign nodes

**Figure 2:**
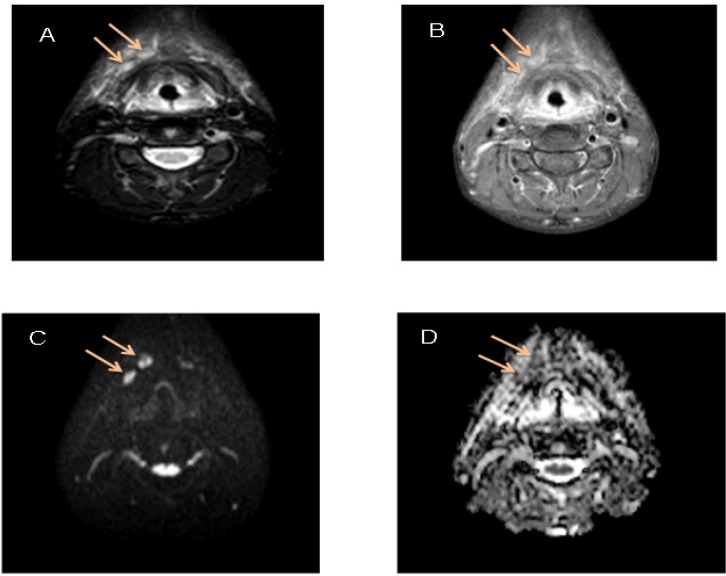
A 17 Year-Old Male with Thyroid Cancer. A, STIR shows two enlarged right neck LNs; B, T1W post contrast shows enhanced LNs; C, DWI shows high signal intensity; D, ADC displays restricted diffusion of LNs on ADC (0.865 × 10^-3^ mm^2^/s)

**Figure 3:**
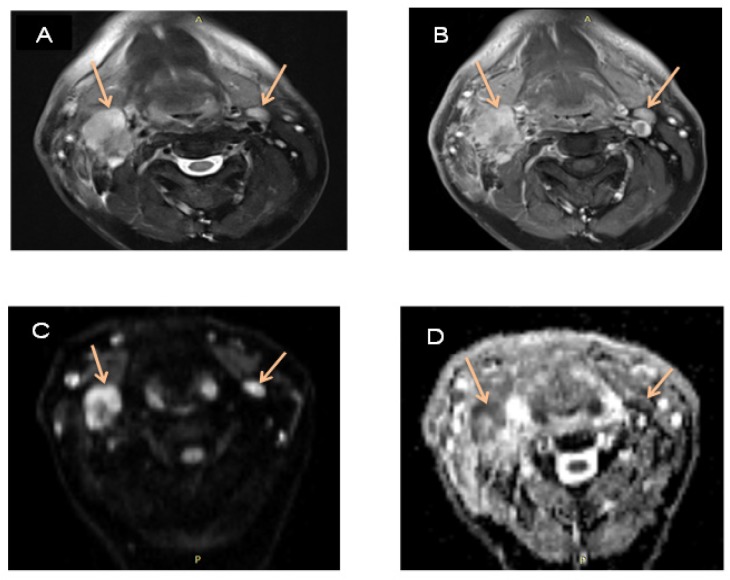
A 45 Year-Old Male with Thyroid and Larynx Cancer. A, T2-W fat sat shows two enlarged right and left neck LNs; B, T1W post contrast shows enhanced LNs; C, DWI shows high signal intensity; D, ADC displays restricted diffusion of LNs on ADC (0.913 × 10^-3^ mm^2^/s)

**Figure 4:**
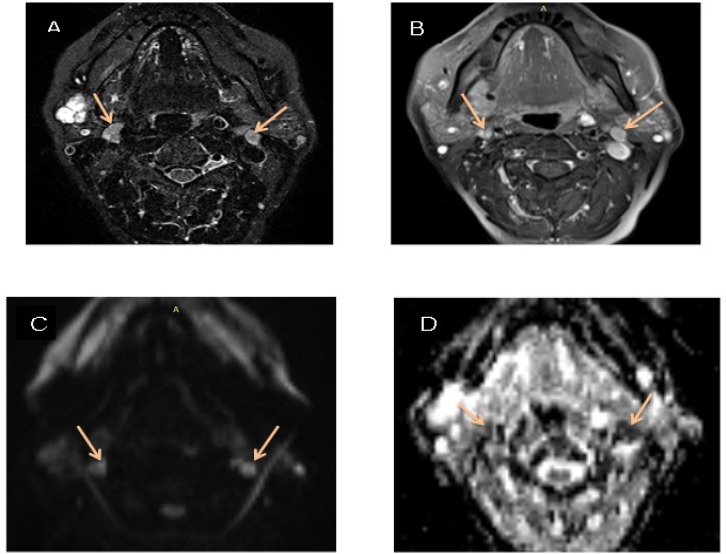
A 63 Year-Old Female with Parotid Mass. A, STIR shows two enlarged right and left neck LNs; B, T1W post contrast shows enhanced LNs; C, DWI shows high signal intensity; D, ADC displays restricted diffusion of LNs on ADC (0.883 × 10^-3^ mm^2^/s)

## Results

Twenty-five patients (14 (56.0%) males, 11 (44.0%) females; mean age, 49.6 years) with 80 malignant and benign lymph nodes were included to the study referring to medical imaging center, located in Shohadaye Tajrish Hospital, Tehran, Iran between May 2017 and January 2018 (Figure 2, 3 and 4). The mean age of patients with benign and malignant tumors was equal to 51.70 and 46.97, respectively. 41.7% of patients with benign tumors and 69.2% of patients with malignant tumors were male. The groups were similar in terms of age and gender (Table 1). 

The mean short-axis diameter of the lymph nodes with metastasis was equal to 7.36 ± 1.55 mm and for the non-metastasis was equal to 7.02 ± 1.23 mm. This difference was not statistically significant considering p-value = 0.346.

Upon pairwise comparison, the mean ADC values (ADCmin, ADCmax, and ADCmean) of benign tumors were significantly higher than the mean ADC values (ADCmin, ADCmax, and ADCmean) of malignant tumors (Table 2).

Receiver operating characteristics (ROC) analysis indicated that ADC mean threshold value was lower than any values in the interval [0.9936 - 0.9999 ×10^-3^ mm^2^/s] that may differentiate malignant lesion from benign lesion (sensitivity by 80.0%, specificity by 77%, area under the curve (AUC) by0.79). Also, ADCmin threshold was any values in the interval [0.8890 - 0.9099 ×10^-3^ mm^2^/s] (sensitivityby100.0%, specificity by 64.0%, AUC by 0.82) and ADCmax threshold was any values in the interval [1.1199 – 1.1100 ×10^-3^ mm^2^/s] (sensitivity by 80.0%, specificity by 77.0%, AUCby0.79) (Figure 1).

## Discussion

Predicting the status of cervical lymph nodes is crucial for diagnosis of malignancy, staging, therapy planning and follow-up in patients with Head and Neck cancer. While conventional imaging can detect enlarged neck lymph nodes, the differentiation between benign and malignant lymph nodes remains challenging as none of the morphological criteria, including size, shape or presence of necrosis is absolutely reliable( Ali, 2012; Ali and El Hariri, 2017; Perrone et al., 2011). 

In our study, the difference in the short axis diameter between metastatic (7.36 ± 1.55 mm) and benign nodes (7.02 ± 1.23 mm) is not statistically significant and there is an overlap in the diameter of the short axis between both groups (p-value = 0.346) that was similar to results of other studies (de Bondt et al., 2009; ElSaid et al., 2014). De Bondt (2009) and ElSaid (2014) stated, there is no significant relationship between the size of the lymph nodes and the presence of metastasis. Hence, the differentiation between benign and malignant neck lymph nodes is not reliable through investigating morphological criteria such as the short axis diameter of lymph nodes (ElSaid et al., 2014). 

In general, invasive methods such as: biopsy and dissection, and their side effects, as well as the low accuracy of other diagnostic imaging methods, such as ultrasound, CT scan, conventional MRI, and limitations of SPECT and PET imaging all have led researchers to make more effort in order to find a method that reduces the time, cost, and complicated diagnostic risks (Razek et al., 2006; Holzapfel et al., 2009; Salem et al., 2014; Liang et al., 2017).

Diffusion-weighted imaging probes local tissue microstructure reflected by the freedom of microscopic motion of water molecules. This motion depends on the size, the temperature, and in particular on the microscopic environment of the examined molecules. So, DWI is an unenhanced, simple, fast and non-invasive MRI technique that can provide information about characterization of LNs and differentiate benign LNs from malignant ones (Perrone et al., 2011; Zhang et al., 2013; Ragheb et al., 2014). 

ADC is a quantitative parameter that is acquired through DWI images and can exclude the T2 shine-effect, also provide quantitative assessment of water diffusivity in the target tissues which makes it easier to differentiate between lesions (ElSaid et al., 2014; Salem et al., 2014; Hasanzadeh et al., 2017).

Similar to studies of (ElSaid et al., 2014; Ali and El Hariri, 2017) we also used high b-value of 1000 s/mm^2 ^to evaluate water diffusion more accurately and decrease the effects of capillary perfusion. 

Our study included 25 patients with 80 enlarged neck LNs. They were 44 patients with benign lymphadenopathy, 36 patients with metastasis from head and neck cancer. The mean ADC values of the benign and metastasis groups were equal to 1.06 ± 0.12 × 10^-3^ mm^2^/s and 0.90 ± 0.10 × 10^-3^ mm^2^/s, respectively. The mean ADC values of the benign cervical lymph nodes were significantly higher than metastatic type that was similar to results of other studies ( Ali, 2012; Dawood et al., 2014; Razek et al., 2006; Salem et al., 2014; Liang et al., 2017; Perrone et al., 2011).

Ali et al., (2012) reported that the mean ADC value of metastatic lymph nodes (0.92 ± 0.13 × 10^-3^ mm^2^/s) was significantly lower than mean ADC of benign cervical lymph nodes (1.51 ± 0.36 × 10^-3^ mm^2^/s) (p< 0.0001).

Dawood et al., (2014) reported that ADC values of benign lymph nodes were significantly higher than those of malignant LNs, with a p value < 0.0001 and the mean ADC value for malignant lesions, that was equal to 0.90 ± 0.15 × 10^-3^ mm^2^/s, which is lower than that of benign LNs, that was equal to 1.52 ± 0.37 × 10^-3^ mm^2^/s.

Razek et al., (2006) reported also a significantly higher mean ADC value in benign nodes (1.64 ± 0.16 × 10^-3^ mm^2^/s) compared to those of malignant nodes (1.09 ± 0.11× 10^-3 ^mm^2^/s ). Similar results was achieved by Perrone (2011) and they reported mean ADC values malignant lymph nodes by 0.85×10^-3^ mm^2^/s versus 1.448× 10^-3^ mm^2^/s for benign nodes. 

Few studies, such as Sumi et al., (2003) reported opposite results; in their studies, the ADC value of metastatic neck LNs was higher than that of non-metastatic one. These results may be explained by the difference in the histological types, the variation in lymph nodes at the cellular level, varying b-values and also heterogeneous ROI. An ROI may also include the necrotic and metastatic cells such that the central necrosis in examined metastatic lymph nodes (48%) altered the ADC values of these nodes; and although low b-value, increases SNR, but reduces the diffusion sensitivity( Ali and El Hariri, 2017; Razek et al., 2006; Holzapfel et al., 2009; Perrone et al., 2011; Hasanzadeh et al., 2017). 

In this study, we also measured the Min and max ADC values. Similarly to the results for the ADC mean, Min and max ADC values of metastatic neck LNs (0.78 ± 0.08 × 10^-3^ mm^2^/s and 1.02 ± 0.12 × 10^-3^ mm^2^/s respectively) were lower than those of non-metastatic neck LNs (0.92 ± 0.20× 10^-3^ mm^2^/s and 1.24 ± 0.15 × 10^-3^ mm^2^/s respectively).

In our study, the best cutoff value of ADC for distinguishing benign from malignant neck lymph nodes was equal to 0.996 × 10^-3^ mm^2^/s with sensitivity, specificity and accuracy by 80.56 %, 77.27 % and 71.59 % respectively, which is approximately similar to the results of the studies which were conducted by de Bondt et al., (2009), Perrone et al., (2011), Taha Ali et al.,(2017), Elsaid et al.,(2014).

The corresponding value in the report of the study which was conducted by de Bondt et al., (2009) was equal to 1.0 × 10^-3^ mm^2^/s with sensitivity and specificity by 92.3 and 83.9 respectively. Perrone et al., (2011) reported that the best cutoff value for discriminating malignant from benign nodes was equal to 1.03 × 10^-3^ mm^2^/s, obtaining a sensitivity by 100% and a specificity by 92.9%. The best ADC threshold value in the study by Elsaid (2014) was equal to 1.005 × 10^-3^ mm^2^/s with sensitivity by 100% and specificity by 62.5% for distinguishing benign from malignant neck lymph nodes. Taha Ali et al., (2017) reported that the optimal ADC threshold value was equal to 1.15 × 10^-3^ mm^2^/s with sensitivity and specificity by 91.6% and 77.7%, respectively for discriminating malignant from benign nodes. 

In general, various studies obtained different values of mean and cutoff ADC for differentiating malignant from benign neck lymph nodes. The difference in these values can be explained by the fact that the ADC is influenced by various factors such as: different MR machines, MRI acquisition parameters, location, size and area of ROI, size of the lymph nodes as well as the body temperature (de Bondt et al., 2009; Perrone et al., 2011; Hasanzadeh et al., 2017).

Our study had some limitations. First: The DW-MRI method, due to spatial resolution limitations, has a low sensitivity in detecting lymph nodes which are less than 5 mm. Therefore, in this study, neck lymph nodes which were less than 5 mm were not evaluated. In order to improve the resolution of diffusion images and achieve more anatomical details of the lesion and to examine lymph nodes which are smaller than 5 mm, the study was suggested to use 3.0 T MRI. Second: trying to improve the diffusion sensitivity by increasing the b value leads to the decline of the signal-to-noise ratio that limits the ADC measurement on the smaller lymph nodes. Third: DWI is sensitive to artifacts such as: motion, susceptibility and chemical shift which makes it difficult to discover lesions on DWI. A further limitation was the low number of patients included in the sample. Further studies can report better and more accurate results regarding DW-MRI, by choosing more samples. 

In conclusion, the results of this study showed that MR diffusion imaging and ADC values as a non-invasive technique can assess metastatic neck LNs in head and neck cancer with higher sensitivity, specificity and accuracy. 

## Conflict of Interest

The authors do not have any possible conflicts of interest.
